# Filter made of cuprammonium regenerated cellulose for virus removal: a mini-review

**DOI:** 10.1007/s10570-021-04319-2

**Published:** 2021-11-23

**Authors:** Shoichi Ide

**Affiliations:** Planova Production Department, Bioprocess Division, Asahi Kasei Medical Co. Ltd, Asahi-machi, Nobeoka, Miyazaki 882-0847 Japan

**Keywords:** Virus removal, Filter, Porous membrane, Cuprammonium cellulose

## Abstract

In 1989, Asahi Kasei commercialized a porous hollow fiber membrane filter (Planova™) made of cuprammonium regenerated cellulose, making it possible for the first time in the world to “remove viruses from protein solutions by membrane filtration”. Planova has demonstrated its usefulness in separating proteins and viruses. Filters that remove viruses from protein solutions, i.e., virus removal filters (VFs), have become one of the critical modern technologies to assure viral safety of biological products. It has also become an indispensable technology for the future. The performance characteristics of VFs can be summarized in two points: 1) the virus removal performance increases as the virus diameter increases, and 2) the recovery rate of proteins with molecular weights greater than 10,000 exceeds the practical level. This paper outlines the emergence of VF and its essential roles in the purification process of biological products, requirements for VF, phase separation studies for cuprammonium cellulose solution, comparison between Planova and other regenerated cellulose flat membranes made from other cellulose solutions, and the development of Planova. The superior properties of Planova can be attributed to its highly interconnected three-dimensional network structure. Furthermore, future trends in the VF field, the subject of this review, are discussed.

## Introduction

Commercial regenerated cellulose fibers are well-known as Viscose rayon, Cuprammonium rayon, and Lyocell fiber. Cuprammonium rayon is produced from cuprammonium cellulose solution. The cuprammonium rayon, famous for “Bemberg®”, is manufactured by Asahi Kasei Corporation, which is now the only company to produce it worldwide. Asahi Kasei introduced this Bemberg technology from J.P. Bemberg of Germany and began manufacturing it in 1931 (Ittou 2007). Hank spinning, continuous spinning, and Net Process (NP)-type spinning methods were developed for Bemberg (Tsurumi 1991). In addition, Asahi Kasei launched a commercial hollow fiber membrane filter for hemodialysis made of cuprammonium regenerated cellulose in 1975. It was derived with regenerated cellulose used for Bemberg, which is a fiber that makes the textile that eventually becomes clothing.

In 1981, Manabe and Iwata et al. discovered that liquid–liquid phase separation in cuprammonium cellulose solution was caused using ketones as nonsolvent, and based on this discovery, porous regenerated cellulose membranes with the mean pore diameter ranging from 10 to 100 nm were made as flat sheet membranes (Manabe et al. 1981). By combining this liquid–liquid phase separation technology in cuprammonium cellulose solution with cellulose hollow fiber membrane manufacturing technique cultivated in artificial kidneys, Asahi Kasei commercialized porous hollow fiber membrane filter (Planova) made of cuprammonium regenerated cellulose, which enabled "virus removal from protein solution by membrane filtration" for the first time in the world in 1989. Filters that remove viruses from protein solutions, i.e., virus removal filters (VFs), have become one of the critical modern technologies to assure a robust viral safety of biological products. It has also become an indispensable technology for the future. Since its practical use in the early 1990s, it has been widely used worldwide as one of the powerful means of virus removal/inactivation in the purification process of plasma-derived medicinal products and biopharmaceuticals (Junter and Lebrun 2017; Inouye and Burnouf 2020; Roth et al. 2020). Planova established its position as a robust and efficient virus removal filter in the VF market, especially in the purification processes of plasma fractionation products. However, due to the large volume of solution to be filtered in the purification process of biopharmaceuticals, VFs made of synthetic polymer membranes, which can provide high filtration pressure, are often used. This reputation is because Planova has a weakness in that its upper limit of filtration pressure is lower than that of VFs made of synthetic polymeric membranes.

In biopharmaceutical purification processes, the application of integrated continuous processing has become a significant trend making it optimal for manufacturing in terms of process and cost considerations (Pollock et al. 2017; Zhang et al. 2017; Fisher et al. 2019). These movements will result in higher concentrations of product intermediates and longer operating times of each unit operation.

This paper begins with the viral safety of biological products and the requirements for VF to introduce the emergence of VF and its essential roles in the purification process of biological products. The comparison between Planova and other regenerated cellulose flat membranes made from other cellulose solutions is described following the phase separation studies for cuprammonium cellulose solution. Further, it runs into the development of Planova. Finally, future trends in the VF field, the subject of this review, are discussed.

### Viral safety of biological products

The representative viruses are listed in Table 1. Viruses vary physical structure (e.g., size, lipid envelope presence), genome structure (RNA/DNA), and resistance to physical/chemical treatment (characteristics of virus family).

**Table 1:** Representative viruses

Virus	Family	Genome	Envelope	Size (nm)
Parvovirus B19, Porcine parvovirus (PPV)	Parvoviridae	DNA	No	18–24
Hepatitis A Virus (HAV)	Picornaviridae	RNA	No	25–30
Polio virus	Picornaviridae	RNA	No	ca.30
Hepatitis B Virus (HBV)	Hepadnaviridae	DNA	Yes	42
Simian virus 40 (SV40)	Polyomaviridae	DNA	No	ca.45
Bovine viral diarrhea virus (BVDV)	Togaviridae	RNA	Yes	40–60
Reovirus 3	Reoviridae	RNA	No	ca.75
Epstein-Barr virus (EBV)	Herpesviridae	DNA	Yes	80–100
Murine leukemia virus (MuLV)	Retroviridae	RNA	Yes	ca. 90
Human immunodeficiency virus (HIV)	Retroviridae	RNA	Yes	ca.100
Human coronavirus	Coronaviridae	RNA	Yes	80 ~ 220
Ebola virus	Filoviridae	RNA	Yes	80 × 800

VFs are used in the purification process of plasma-derived medicinal products and biopharmaceuticals to contribute viral safety to these products. Plasma-derived medicinal products are immunoglobulins (IgG), coagulation factors such as Factor VIII, Factor IX, prothrombin complex, and inhibitors purified using plasma as a raw material. These products have the risk of potential viral contamination because they are derived from human blood. On the other hand, biopharmaceuticals are produced from cell lines of human or animal origin cell lines utilizing genetic recombination technology or cell fusion technology. These products include monoclonal antibodies, recombinant proteins, vaccines, etc. Biopharmaceuticals are expressed in cell lines such as CHO cells. There may be endogenous retroviruses and non-infectious retrovirus-like particles in these cells. In addition, methods should address the risk of contamination with adventitious viruses that may introduce in the culture process or subsequent purification process. Virus removal filters are used in biopharmaceutical processes to remove both endogenous and exogenous viruses.

Virus clearance methods for plasma-derived medicinal products and biopharmaceuticals have been taken globally since the early 1990s around the time of some infection accidents caused by plasma-derived medicinal products. In the document entitled *Guideline on plasma-derived medicinal products* (1996), European Medicines Agency/CHMP Guidelines (CPMP/BWP/269/95: Note for guidance on plasma-derived medicinal products) as a guideline for viral safety of plasma-derived medicinal products, required incorporating multiple orthogonal methods for virus clearance with independent mechanisms and process validation of virus clearance. World Health Organization (WHO) guideline (2004) for viral safety of blood plasma products was also issued. On the other hand, for biotechnology products, ICH(International Conference on Harmonization) Q5A (1997) issued "Viral safety evaluation of biotechnology products derived from cell lines of human or animal origin ", following the ICH guidelines, it led to the establishment of guidelines of FDA/CBER (1997), European Medicines Agency/CHMP/BWP P (2008) and Ministry of Health and Welfare (2000) regulatory. The approach to ensuring viral safety presented in these guidelines is mainly composed of the following:

- screening cell lines and other raw materials for absence of viruses
- assessing the ability of virus removal/inactivation in manufacturing steps
- testing the product at appropriate stages of production for absence of viruses

The virus removal filter plays a role in b) described above.

Currently, virus removal and inactivation methods introduced in the manufacturing process of biological products include the following: as virus inactivation methods, there are 1) heat treatment, 2) low pH incubation, 3) chemical treatment such as S/D (Solvent-Detergent), 4) irradiation treatment, etc., and as virus removal methods, there are 5) membrane filtration (virus removal filtration), 6) chromatography, and 7) precipitation. In the manufacturing process, multiple orthogonal methods for virus clearance with independent mechanisms are used in combination (CPMP/BWP/269/95; Aranha 2001a, b). Virus removal filtration is de facto standard as a common unit operation and illustrated as a robust and safe virus removal technology for biological products, due to the size exclusion mechanism. A solvent-detergent inactivation method is effective against only enveloped viruses. For the heat treatment inactivation method, it is known that heat-resistant viruses exist within a population of viruses. The VF method is effective against all larger viruses than the membrane’s pore size without recourse to the family and genome of viruses. Also, the VF method does not lead to denaturing effects of proteins, and the performance of VF has less influenced by process conditions (Aranha 2001a, b). The stream for viral safety of biological products is summarized in Table 2.

**Table 2:** Stream for viral safety of biological products

Year	Inactivation/Removal	Method	Remark	Regulation
1941	Removal	Precipitation	Cohn method, Partitioning of proteins, Some virus removal	
1970s	Removal	Ion Chromatography	Some virus removal	
1980s	Inactivation	Pasteurization	Risks of protein denaturation	
	Inactivation	Dry-heat treatment	Risks of protein denaturation	
	Inactivation	Low pH incubation	Restricted to immunoglobulins, Limited inactivation of non-enveloped vireses	
1985	Inactivation	Solvent-detergent (S/D)	Inactivation of only enveloped viruses	
1989	Removal	**Virus removal filter** (VF)	Robust, No restricted to types of viruses, Size exclusion, No protein denaturation	
1990s				**Requirement for incorporating multiple orthogonal methods** 1996 CPMP for Plasma-derived products, 1997 ICH for biotechnology products, 1997 FDA for monoclonal antibody, 2001 MHW for biotechnology products

### Requirements for VF

Based on the principles of size exclusion, VF needs to remove viruses in the membranes and permeate proteins through the membranes. VFs must overcome the technical difficulty of separating particles that do not differ significantly in sizes, such as 20–100 nm for viruses and several nm to a dozen nm for proteins.

The performance required for VF is as follows.

1. Virus removal performance: rejection rate of 99.99% or higher (LRV of 4 or higher).
2. Protein recovery rare: 90% or more.
3. Slight decrease in these performances during filtration.

Virus removal performance is expressed in log removal rate, *i.e.*, LRV (Logarithmic Reduction Value). When the concentrations of the viruses before and after the filtration are N_0_ and N_f_, respectively, LRV = log_10_(N_0_/N_f_) is expressed. The permeation performance of the proteins is evaluated by the permeability(L/hr) and recovery rate (%) of the proteins, the integrated permeation volume(L/m^2^/hr) and the integrated permeation weight (kg/m^2^/hr) of the proteins, etc. Ultimately, it is reflected in the membrane area required for filtration, i.e., the filter cost.

### Phase separation studies for the cuprammonium cellulose solution

The regeneration of the cellulose solution by coagulation with non-solvent is a vital pathway to transform native cellulose into valuable materials in various forms, such as fibers, films/membranes, beads/microspheres, hydrogels/aerogels, bioplastics, etc*.* (Wang et al. 2016). These regenerated cellulose fibers and membranes are still manufactured by viscose and cuprammonium processes.

**Fig. 1:**
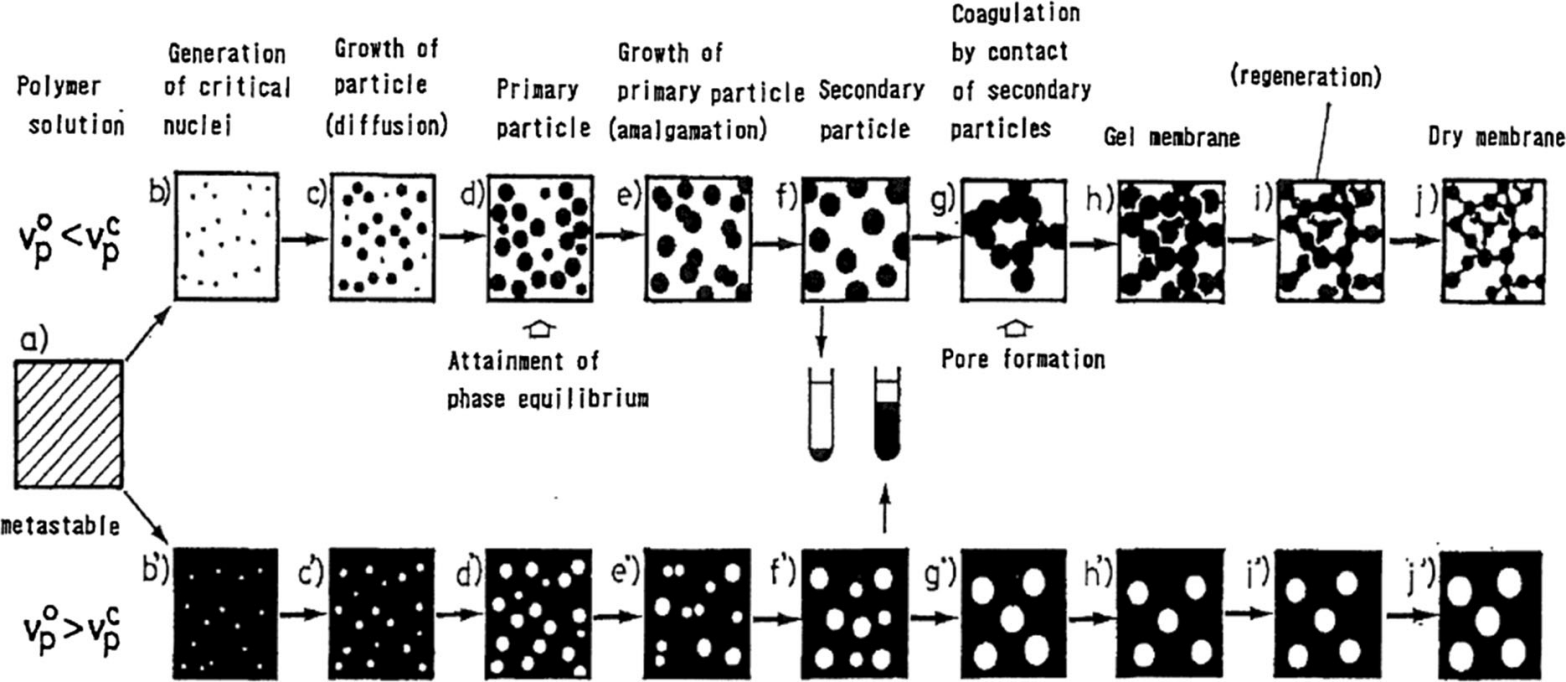
Elementary steps in porous membrane formation by the phase separation method: v_p_^0^,the initial polymer volume fraction of the solution when the phase separation occurs; v_p_^c^, polymer volume fraction at a critical solution point (Kamide et el. 1994). Copyright 1994 The Society of Polymer Science, Japan

In 1981, Manabe and Iwata et al. discovered that liquid–liquid phase separation in cuprammonium cellulose solution was caused using ketones as non-solvent. Based on this discovery, porous regenerated cellulose membranes with the mean pore diameter ranging from 10 to 100 nm were made as flat sheet membranes (Manabe et al. 1981). Kamide and Manabe noticed the importance of the “particle growth concept” for membrane formation mechanism in the non-solvent induced phase separation method. They observed primary particles, growth from primary particles to secondary particles, and secondary particles by electron microscopy during the phase separation process of a system in which a cuprammonium cellulose solution was coagulated with acetone, ammonia, and aqueous solution (Kamide and Manabe 1985). The elementary steps of porous membrane formation by the phase separation method is shown in Fig. 1. When the initial polymer concentration of the polymer solution v_p_^0^ is smaller than the polymer concentration at a critical solution point v_p_^c^, the polymer-rich phase forms first as the nuclei and then separates as primary particles. The primary particles coalesce into larger secondary particles. Subsequently, the secondary particles contact each other to form a gel membrane, which undergoes regeneration and drying to become a dry membrane. A highly interconnected pore structure is formed due to the aggregation of the nodules, *i.e.*, the secondary particles (Kesting 1985, 1990; Kamide 1990; Kamide et al. 1993; van de Witte et al. 1996). A membrane formed under v_p_^0^ < v_p_^c^ has non-circular pores.

Manabe et al. investigated some morphological characteristics of regenerated porous cellulose flat membrane and cellulose acetate flat membranes prepared by phase separation method, (1) to evaluate the pore structure of the membranes by the electron micrographic method and (2) to explain the morphological characteristics of these membranes in light of the development of the phase separation of the solution during the casting process (Manabe et al. 1987). They evaluated the transport phenomena of ions and non-electrolyte molecules through an interfacial boundary between the casting solution and the coagulating solution in the process of forming the membranes. The regenerated porous cellulose flat membrane was prepared from cuprammonium cellulose solution by coagulating with the acetone-ammonia-water solution. They noted that when the phase separation occurs for the cellulose-copper-ammonia-water–acetone system, copper, ammonia, and acetone concentrate in the polymer-rich phase, while water molecules remain in the polymer-lean phase. They concluded that when the total flux of water and ammonia from the casting solution to the coagulation solution is always larger than the total flux of acetone from the coagulation solution to the casting solution, the mean pore diameter and porosity decrease with distance (Z) from the surface.

Kamide et al. confirmed the particle growth of non-solvent induced phase separation in the cuprammonium cellulose solution/coagulating solution system by dynamic light scattering measurement. In the polymer solution/coagulating solution system, *i.e*., cuprammonium cellulose solution/acetone-ammonia-water solution and cuprammonium cellulose solution/sodium hydroxide-water solution, the particle size distribution and the number-average radius of the growing particles were evaluated (Kamide et al. 1994). They found that liquid–liquid phase separation occurred in both the cuprammonium cellulose solution/acetone-ammonia-water solution and the cuprammonium cellulose solution/sodium hydroxide-water solution system.

Inamoto et al. explored the morphological formation of the regenerated cellulose flat membranes made from cuprammonium cellulose solution using various aqueous coagulants (H^+^, Na^+^, K^+^, NH_4_^+^, Ca^2+^, Mg^2+^ with various counter ions). Those results indicated that the morphology in the membranes is mainly categorized into four types depending on pH and cation species of coagulating solution and controlled by complex forms of the coagulated gel (Inamoto et al. 1996). The above results indicated that liquid–liquid phase separation occurs in the system of cuprammonium cellulose solution/various aqueous coagulants.

**Fig. 2:**
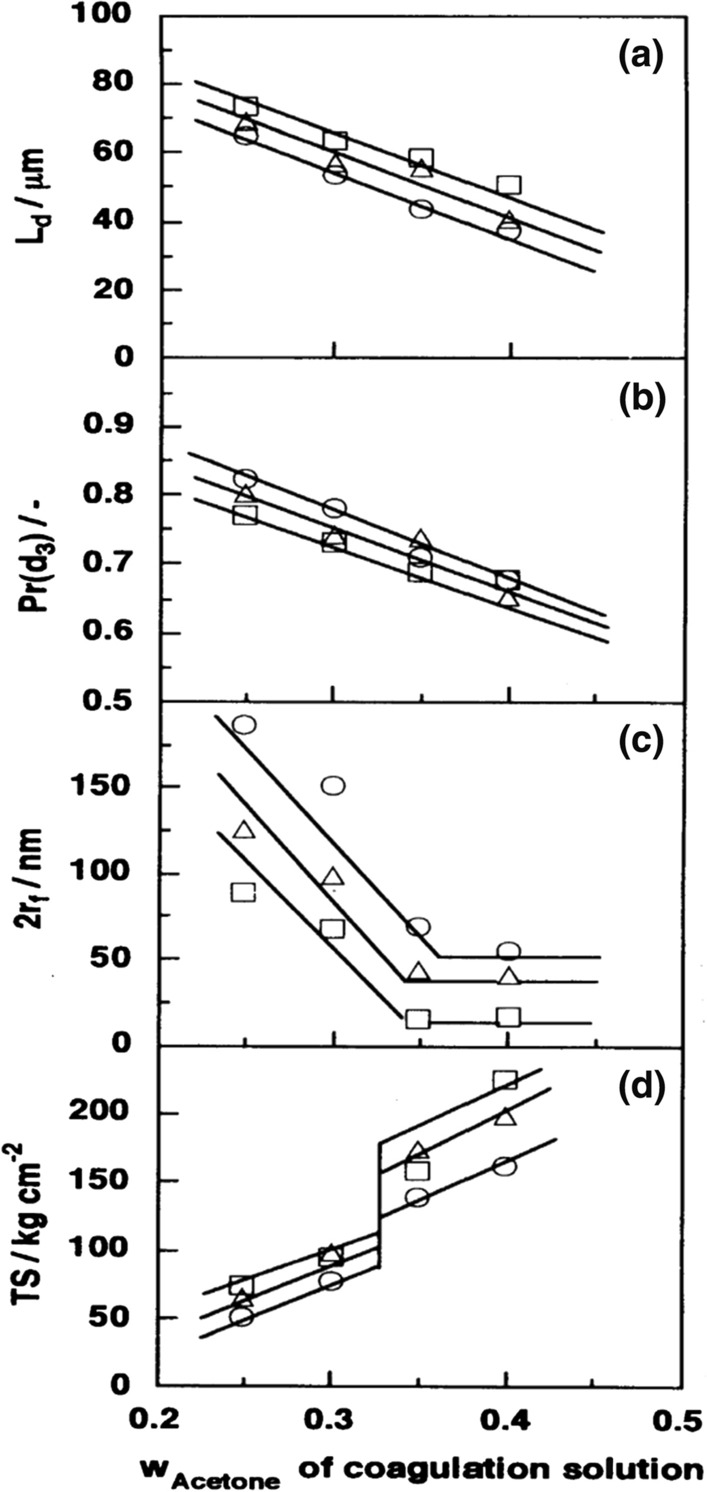
Effects of weight faction of acetone (w_Acetone_) of the coagulation solution on the thickness of dry membrane L_d_ (a), porosity Pr(d_3_) (b), mean radius of pores 2r_f_(c), and tensile strength (TS) of the membranes(d) (Iijima et al. 1997). Copyright 1997 The Society of Polymer Science, Japan

Iijima et al. investigated phenomenological effects of non-solvent induced phase separation conditions on pore characteristics of porous regenerated cellulose membranes, that is, flat membranes made by using cuprammonium cellulose solutions and aqueous acetone solutions as coagulation solution. For example, Fig. 2 indicates changes in membrane thickness of the dry membrane, membrane porosity, pore diameter measured by the water-flow-rate method, and tensile strength of the membranes, prepared using coagulation solutions with different acetone concentration. Figure 2c shows that 2r_f_ decreases inversely linear relation to W_Acetone_ in regions W_Acetone_ < 0.35, whereas 2r_f_ is kept almost constant in regions W_Acetone_ > 0.35. They mentioned that the dramatic change in the W_Acetone_ dependence of 2r_f_ at W_Acetone_ = 0.35 closely corresponds to the change in pore shape from noncircular to circular, which consists of the “particle growth concept”. The pore shape depends on the polymer concentration relative to the critical solution point for polymer solution/coagulating solutions (Iijima et al. 1997).

Cao et al. studied the preparation and properties of regenerated microporous cellulose flat membranes prepared by coagulation of cellulose/NaOH solution with aqueous H_2_SO_4_ solution. The obtained membrane showed an asymmetric porous structure. The membranes’ average pore diameter (2r_f_) values measured by the filtration velocity method ranged from 32.4 to 39.5 nm. The water flux (*J*) of the membranes ranged from 7.2 to 9.69 (ml/cm^2^/h/0.1 MPa) (Cao et al. 2006). Mao et al. prepared regenerated cellulose flat membranes from cellulose/NaOH/urea aqueous solution by coagulating with various coagulants including H_2_SO_4_, HOAc, H_2_SO_4_/Na_2_SO_4_, Na_2_SO_4_, (NH_4_)_2_SO_4_, H_2_O, C_2_H_5_OH, and (CH_3_)_2_CO, respectively, and the effect of coagulants on the porous structure was investigated. The 2r_f_ values of the obtained membranes measured by the filtration velocity method ranged from 25.7 to 56.8 nm. The water flux (*J*) of the membranes ranged from 32.76 to 198.5 (ml/m^2^/h/mmHg), and the rejection rate of bovine serum albumin (BSA: molecular weight 67,000) to the membranes was evaluated (Mao et al. 2006).They suggested that the pure water flux of the membranes was related mainly to the interior structure of the membranes (Mao et al. 2006). Liu et al. investigated the properties of regenerated cellulose flat membranes prepared from cellulose/LiOH/urea aqueous solution by coagulating with aqueous H_2_SO_4_ solutions. The 2r_f_ values of the obtained membranes measured by the filtration velocity method ranged from 21.2 to 52.1 nm. The water flux (*J*) of the membranes ranged from 8.4 to 11.2 (ml/m^2^/h/mmHg) (Liu et al. 2010). Zhang et al. studied the formation and properties of regenerated cellulose flat membranes made from cellulose/*N*-Methylmorpholine-*N*-oxide (NMMO) solution by coagulating with water and NMMO/water solutions. The 2r_f_ values of the obtained membranes measured by the filtration velocity method ranged from 10.94 to 41.53 nm. The water flux of the membranes ranged from 2.5 to 12 (ml/cm^2^/h/0.2 MPa). The rejection rate of bovine serum albumin (BSA: molecular weight = 67,000) to the membranes was from 10 to 90% (Zhang et al. 2001). These results indicate that membranes with an average pore size of 10–100 nm can be produced by liquid–liquid phase separation even when regenerated using cellulose solutions other than cuprammonium cellulose solution.

Here, Table 3 compares membrane properties between Planova 35N hollow fiber membranes made from cuprammonium cellulose solution (described below) and the flat membranes described above made from other cellulose solutions. By comparing membranes with an average pore size of about 35 nm, it can be said that the water flux (J) of Planova 35N is 3 to 40 times higher than that of membranes prepared from other cellulose solutions. The albumin recovery rate of Planova 35N is also much higher than that of other membranes. The superior properties of Planova 35N can be attributed to its highly interconnected three-dimensional network structure, which will be discussed later. In other words, there are few closed pores and half-closed pores in Planova.

**Table 3:** Comparison of the membrane properties between Planova 35N prepared from cuprammonium cellulose solution and the membranes prepared from other cellulose solutions

Membrane	Polymer Solution	Coagulant	2r_f_ (nm)	*J* (mL/m^2^/hr/mmHg)	φ(%)
Planova 35N	Cellulose/cuprammonium	Acetone/NH_3_/H_2_O	35	330	100
Cao et al. (2006)	Cellulose/NaOH aq	H_2_SO_4_ aq	35.7	98	NA
Mao et al. (2006)	Cellulose/NaOH/urea aq	5% H_2_SO_4_/ 5%Na_2_SO_4_ aq	34.8	50.3	> 95
Same as above	Same as above	H_2_O	34.4	42.9	> 95
Same as above	Same as above	Acetone	25.7	33.8	85.2
Liu et al. (2010)	Cellulose/LiOH/urea aq	5% H_2_SO_4_ aq	33.5	8.8	NA
Zhang et al. (2001)	Cellulose/NMMO aq	H_2_O	31.0	17	18
Same as above	Same as above	22.5% NMMO aq	23.2	27	30

2r_f_: average pore diameter measured by the filtration velocity method (nm)

*J*: water flux per unit membrane area (mL/m^2^/h/mmHg)

φ: recovery rate of bovine serum albumin (%)

## Development of Planova

### Concept

As described above, Manabe and Iwata et al. discovered that liquid–liquid phase separation in cuprammonium cellulose solution was caused using ketones as nonsolvent (Manabe et al. 1981). By combining this liquid–liquid phase separation technology in cuprammonium cellulose solution with cellulose hollow fiber membrane manufacturing technique cultivated in artificial kidneys, Asahi Kasei commercialized porous hollow fiber membrane filter (Planova) made of cuprammonium regenerated cellulose, which enabled "virus removal from protein solution by membrane filtration" for the first time in the world in 1989.

**Fig. 3:**
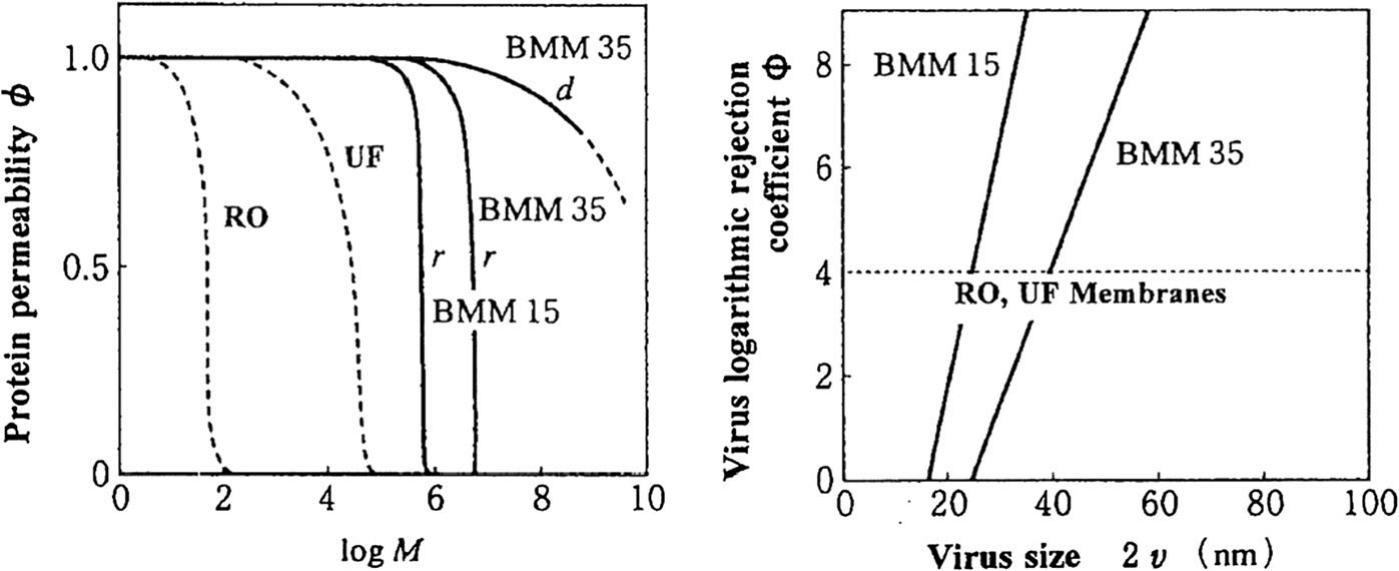
Schematic representation of relationships between protein permeability (φ) and protein molecular weight (M) and relationships between virus LRV (Φ) and virus size (2v) for BMM15, BMM35, RO and UF (Manabe 2003). Copyright 2003 The Mass Spectrometry Society of Japan

Extreme sharp fractionation performance of the membranes is required to meet the requirements of VF. The performance characteristics of VF can be summarized in two points: 1) the virus removal performance increases as the virus diameter increases, and 2) the recovery rate of proteins with molecular weights greater than 10,000 exceeds the practical level (Manabe 1992, 2003). A schematic representation of the relationships between protein permeability (φ) and protein molecular weight (M) and the relationships between virus LRV (Φ) and virus size (2v), comparing VF with RO and UF filters, is shown in Fig. 3. Planova is the trade name of Asahi Kasei's filter made of regenerated cellulose for virus removal, and BMM is the name of the hollow fiber membrane composed of Planova. In other words, Planova and BMM are practically the same. In Fig. 3, the virus LRVs of the RO and UF filters do not increase with increasing virus diameter, while the virus LRV of the BMM increases with increasing virus diameter. The virus LRVs of the RO and UF filters do not increase with virus diameter because the RO and UF filters have structural defects that prevent them from removing the viruses. However, the virus LRV of BMM increases with increasing virus diameter. It is attributable to less breakage of the BMM’s pore structure. Since the pore size of RO and UF filters is smaller than the size of proteins, so the protein recovery rate, i.e., the permeability of proteins with molecular weights greater than 10,000 in RO and UF filters, is almost zero, which is not practical. However, with BMM15 and BMM35, the recovery rate (φ) of proteins with molecular weights greater than 10,000 exceeds the practical level, making it possible to permeate proteins of biological products. BMM15 and BMM35 are hollow fiber membranes composing of Planova15N and Planova 35N filters.

### Manufacturing method

**Fig. 4:**
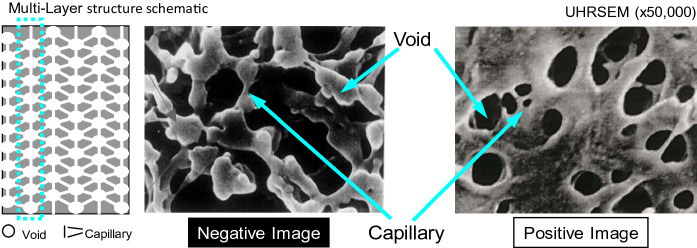
Multi-layer structure schematic and three-dimensional network structure consisting of interconnected “void” pores and “capillary” pores of Planova. (Yokogi and Satoh 1998) Copyright ©1998 Jiho Inc. Copyright ©2021 Asahi Kasei Medical Co., Ltd

**Fig.5:**
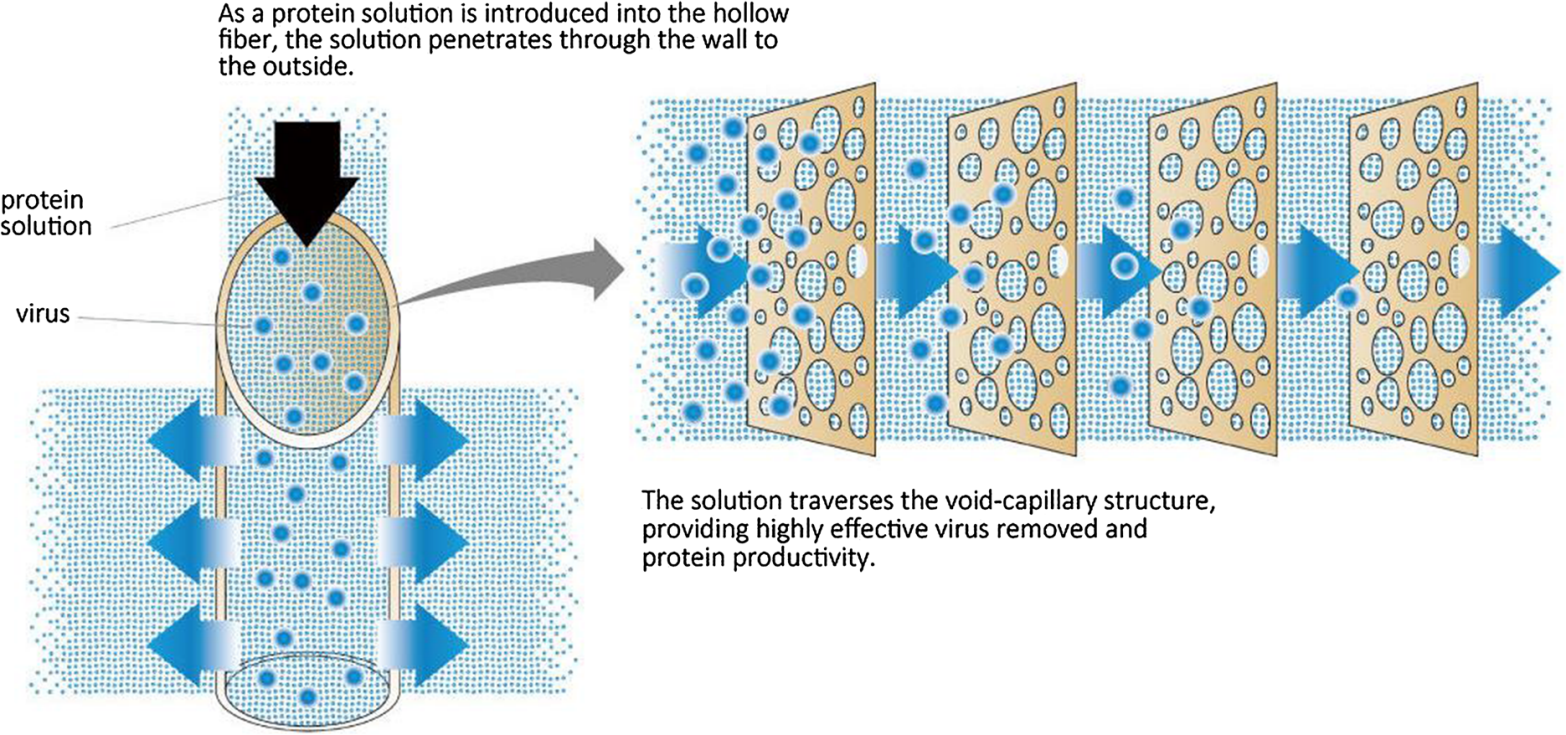
Schematic of size exclusion filtration mechanism. Copyright ©2021 Asahi Kasei Medical Co., Ltd

To make this performance feature for VF, we aimed to realize a multilayered structural membrane. A multilayered structural membrane means a membrane in which a layer functions as a screen layer and the layers are multilayered, as indicated in Fig. 4. Furthermore, the multilayered structure of Planova, as observed using SEM, is also shown in Fig. 4. Planova has a multilayered structure, i.e., a three-dimensional network structure consisting of interconnected “void” pores and “capillary” pores that act as a multilayered structural membrane. The positive image in Fig. 4 is an ordinary photograph using SEM, and the black parts in the positive image are pores. On the other hand, the negative image in Fig. 4 is opposite to an ordinary photograph. This image results from embedding a cellulose membrane with an epoxy resin, then dissolving the membrane with cuprammonium solution and photographing the structure of the remained epoxy resin by SEM (Tsurumi et al. 1990a, b). Namely, the white parts in the negative image, the remaining epoxy resin, are pores. This negative image explicitly indicates the existence of a three-dimensional network consisting of interconnected “void” pores and “capillary” pores in the membrane. The three-dimensional network consisting of “void” pores and “capillary” pores; in other words, bead structure comes from the adequate growth of polymer-rich phase after the phase separation. "U-shaped tubular spinning method" is an invented manufacturing method to achieve this membrane structure (Ide et al. 1991). Namely, by forming a membrane structure with less tension at the coagulating stage, an excellent membrane structure with less breakage of the pore structure can be obtained; the U-shaped tubular spinning method makes the membrane structure a three-dimensional structure with less breakage of the pore structure, which achieves high virus removal and high protein recovery (Tsurumi et al. 1990a, b). Tsurumi et al. investigated the structure of the membranes using an electron microscope. They observed three planes, XY, YZ, and ZX (X is the radial axis, Y is the fiber direction axis, and Z is the peripheral direction axis). Then, to evaluate the multilayer structure, they examined the change in the size of the "capillary" pores with the distance of the thickness of the membrane (X-axis). As a result, it was found that the size of the "capillary" pores was almost the same throughout the membrane thickness (Tsurumi et al. 1990a, b). The schematic of the size exclusion filtration mechanism of Planova is shown in Fig. 5. As a protein solution is introduced into the hollow fiber membrane, the proteins permeate through the membrane to the outside. At the same time, viruses are captured in the interconnected “void” pores and “capillary” pores of the membrane by multilayer filtration.

### Innovation from VF for large viruses to VF for small viruses

**Fig.6:**
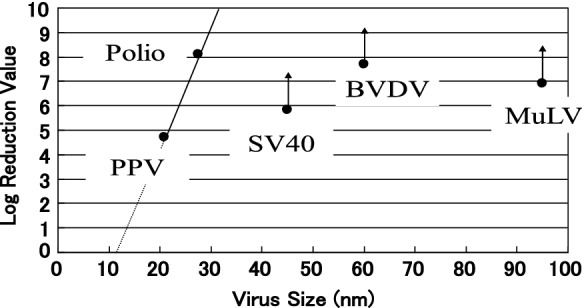
Virus LRV of Planova 20N on various virus size (Ide et al. 2002). Copyright ©2002 Asahi Kasei Medical Co., Ltd

Planova 35N, with a mean pore size of 35 nm, was launched as the first commercial VF all over the world in 1989. Planova 35N were developed specifically for removing are large viruses (such as HIV, > 80–120 nm) and medium viruses (such as hepatitis B virus (HBV), 35 nm). Planova15N, with a mean pore size of 15 nm, was launched in 1992 to target towards small virus (such as, parvovirus B19, 18–26 nm, HAV). However, it is difficult to apply Planova 15N to proteins with relatively large molecules such as IgG and Factor VIII due to their low recovery rate. This is because the diameter of the virus to be removed (approximately 18 nm or more) is too close to the diameter of the protein to be permeated. Planova 20N was launched in 2001 to apply large molecule proteins such as IgG and Factor VIII to overcome this difficulty. Planova 20N shows a higher porcine parvovirus (PPV) LRV in the initial and subsequent filtration stage while passing through IgG, setting the extruding rate and winding rate to constant values to manufacture a hollow fiber membrane can change the membrane structure by adjusting the polymer concentration in the polymer solution, composition of inner coagulation solution, and composition of outer coagulation solution. As Iijima et al. investigated the phenomenological effects of non-solvent induced phase separation conditions on pore characteristics of porous regenerated cellulose flat sheet membranes (Iijima et al. 1997), we explored the effects of non-solvent induced phase separation conditions on pore characteristics of porous regenerated cellulose hollow fiber membranes. By decreasing the non-solvent concentration of both the inner and outer coagulation solution, we accomplish the Planova 20N (Ide and Noda 2000). When the non-solvent concentration in both the inner and outer coagulation solution decreases, the pore size distribution of the region of the membrane that substantially contributes to the virus removal can be narrower, and the thickness of this region can be increased. The virus LRV of Planova 20N on various virus size is shown in Fig. 6 (Ide et al. 2002). Furuya et al. indicated that the virus removal efficiency was noticeably increased in the plasma-derived Factor VIII manufacturing process by changing Planova 35N to Planova 20N, without variation of the biochemical properties or a serious loss of Factor VIII (Furuya et al. 2006). Jorquera reported that intravenous immunoglobulin therapy has been developed through Planova 20N, allowing further viral safety (Jorquera 2009).

### Characteristics of Planova

The characteristics of Planova are mainly composed of the following:1) high virus removability, 2) unrestricted for the virus to be removed, and 3) high protein recovery rate. Planova 20N maintained a high virus clearance of PPV LRV > 4 for PPV-spiked IgG solutions (IgG concentration range 1–30 mg/mL), regardless of viral spike conditions or filtration rate. Furthermore, there was little decay of the filtration flux during filtration. By observing the capture area of gold particles, it has been shown that Planova 20N has a large capacity for virus capture. Planova 20N is a well-balanced, robust, small virus-retentive filter (Hongo-Hirasaki et al. 2006). We investigated the effect of varying virus-spiking conditions on the filter performance (flux, flux decay, PPV LRV) of Planova 20N and observed captured PPV particles inside the membrane using TEM. Planova 20N was shown to be a robust filter unaffected by varying virus-spiking conditions (Hongo-Hirasaki et al. 2011). We investigated the effect of antibody solution conditions (ionic strength, pH, IgG concentration, buffer composition, and aggregate level (dimer content)) on filter performance for a virus removal filtration process using Planova 20N. These results indicated that Planova 20N is applicable for a wide range of solution conditions (Hongo-Hirasaki et al. 2010). As just described, we revealed that the filter performance of Planova 20N was not easily affected by varying virus-spiking conditions and Planova 20N has a well-balanced performance and robust feature.

It is known that membrane fouling occurs during virus filtration. Hamamoto et al. investigated the mechanisms of decline in virus filter performance due to membrane fouling using Planova 20N (Hamamoto et al. 2018). Their analysis showed that the primary cause of flux decline appeared to be irreversible IgG adsorption on the surface of the virus filter membrane. They said that analyses of adsorption and desorption and conformational changes in IgG molecules on cellulose surfaces provide an effective approach for identifying ways of optimizing solution conditions to maximize IgG throughput of virus removal filter. The use of these analyses may have a possibility to improve cellulose-based virus removal filters.

### Pressure release during filtration

It is shown when the pressure is released during filtration and filtration restart again after some time, and the virus could leak into filtrate (Woods and Zydney 2014; LaCasse et al. 2016). This phenomenon can be explained that, during filtration, the Brownian motion of the virus is restricted by hydrodynamic force by the filtration pressure. Still, when the filtration pressure is released, the Brownian motion of the virus becomes active and the virus moves by diffusion (Yamamoto et al. 2014). By this means, it is essential to evaluate more careful optimization of virus removal filtration conditions, especially depressurization. Strauss et al. characterized the impact of pressure on MVM (Minute virus of mice) LRV, and established design spaces to ensure effective small virus removal for Planova 20N (Strauss et al. 2017).

### Observation of capturing virus in the membrane

Yamaguchi et al. analyzed the capturing status of colloidal gold particles and parvovirus B19 in the BMM hollow fiber (BMM15, BMM20, BMM35) which constitutes Planova by immunoelectron microscopy. These results showed that BMM20 and BMM15 could retain microparticles larger than 20 nm, such as colloidal gold particles and parvovirus B19 inside the membrane (Yamaguchi et al. 2007). Adan-Kubo et al. reported that they simultaneously analyze the gross structure of a virus removal filter and visualize virus entrapment during a filtration process conducted under actual manufacturing conditions using Planova 15N and Planova 20N (Adan-Kubo et al. 2019). Ayano et al. proposed a novel method for monitoring of dynamic process of virus capture in a single hollow fiber membrane comprising Planova 20N (Ayano et al. 2021). Various imaging techniques have been used to observe how viruses are trapped in the intricate structure of the membrane filter; however, they are limited to ‘static’ imaging due to the images after filtration. They have succeeded in detailed monitoring of the ‘dynamic process’ of virus-like particles (VLP) capture (Cetlin et al. 2018) in the membrane during filtration using an ultra-stable optical microscope. This observation method contributes to novel insights to create a new virus removal filter and biopharmaceutical manufacturing.

### Removal of emerging viruses

Various emerging viruses as dengue virus, Hepatitis E virus (HEV), and Zika virus have emerged. Several virus clearance studies with emerging viruses were conducted. These results showed that Planova could remove emerging viruses independent of the characters of viruses (Blümel et al. 2017; Yue et al. 2019; Kapsch et al. 2020). As a result, the robust virus removability of Planova was confirmed. In response to the current major social problem of SARS-CoV-2 virus, the development of vaccines and therapeutics using antibodies from plasma and serum obtained from recovered Covid19 patients is carried out vigorously. Virus removal filters are off course used in order to support the early provisions of vaccines and therapeutic agents for Covid19.

## Current and future trends in the field of VF

### Virus removal filters on the VF market

Currently, virus removal filters are classified into two categories based on the size of the viruses to be removed. Large virus retentive filters can remove large viruses (e.g. retroviruses, 80–120 nm) and medium viruses (e.g. HBV, 35 nm). Small virus retentive filters can remove both small viruses (e.g. parvovirus B19, 18–26 nm, HAV, 25–30 nm) and larger than small viruses. The Planova 35N belongs to the large virus retentive filters, while the Planova 15N and Planova 20N are small virus retentive filters. (Parenteral Drug Association (PDA) Technical Report 41 2005). In addition to plasma fractionated products, VFs have been widely used in the purification process of biopharmaceuticals such as monoclonal antibodies and recombinant proteins since around 2010. Planova established its position as a robust and efficient virus removal filter in the VF market, especially in the purification processes of plasma fractionation products. However, due to the large volume of solution to be filtered in the purification process of biopharmaceuticals, VFs made of synthetic polymer membranes, which can provide high filtration pressure, are often used. It is because Planova has a weakness in that its upper limit of filtration pressure is lower than that of VFs made of synthetic polymeric membranes. Therefore, a new Planova with an improved upper limit of filtration pressure is required. Despite this situation, Planova holds a strong position in the VF market. The reasons are as follows: 1) Planova is composed of hydrophilic cellulose, which has the advantage of low protein adsorption; 2) Planova has a three-dimensional network consisting of interconnected "void" pores and "capillary" pores, and the thickness of Planova's multilayered structure, which provides the advantage of low flux decay during filtration and high protein recovery, while also providing high virus removability.

Currently, commercially available virus removal filters are listed in Table 4.

**Table 4:** Virus removal filters on the VF market

Manufacture	Filter	Category of VF	Membrane type	Material
Asahi Kasei	Planova™ 15N	Small virus	Hollow fiber	Regenerated cellulose
	Planova™ 20N	Small virus	Hollow fiber	Regenerated cellulose
	Planova™ 35N	Large virus	Hollow fiber	Regenerated cellulose
	Planova™ BioEX	Small virus	Hollow fiber	PVDF
Merck (MilliporeSigma)	Viresolve® NFP	Small virus	Flat sheet	PVDF
	Viresolve® NFR	Large virus	Flat Sheet	PES
	Viresolve® Pro	Small virus	Flat sheet	PES
Danaher (Pall)	Ultipor® DV20	Small virus	Flat sheet	PVDF
	Ultipor® DV50	Large virus	Flat sheet	PVDF
	Pegasus™ SV4	Small virus	Flat sheet	PVDF
	Pegasus™ Prime	Small virus	Flat sheet	PES
Sartorius Stedim	Virosart® CPV	Small virus	Flat sheet	PES
	Virosart® HC	Small virus	Flat sheet	PES
	Virosart® HF	Small virus	Hollow fiber	PES

*PVDF*: Hydrophilic modified polyvinylidenedifluoride (PVDF)

*PES*: Hydrophilic modified polyethersulfone (PES)

### VF in integrated continuous processing

In biopharmaceutical purification processes, the application of integrated continuous processing has become a significant trend to improve productivity and cost effectiveness (Pollock et al. 2017; Zhang et al. 2017; Fisher et al. 2019). These movements will result in higher concentrations of product intermediates and longer operating times of each unit operation. Lute et al. show that the Planova 20N and Planova BioEX virus filters are capable of effectively removing bacteriophage PP7 (> 4 log) for 10 mg/ml h-IgG for up to 4 days (Lute et al. 2020). A VF suitable for the higher concentration of protein solutions and long filtration time might be needed.

### New usage of VF

Recently, there has been strong attention to gene therapy products, especially those utilizing recombinant adeno-associated viral (rAAV) vectors. The most commonly found impurities in rAAV stocks include defective particles (i.e., AAV capsids that do contain the therapeutic gene or are not infectious), residual proteins from host cells and helper viruses (adenovirus, herpes simplex virus, or baculoviruses), illegitimate DNA, plasmids, cells, or helper viruses. It is so important to remove impurities in the purification process during the manufacturing of rAAV. (Penaud-Budloo et al. 2018; Adams et al. 2020). In this purification process, rAVV vectors were removed from helper viruses using a virus removal filter (Bogedain et al. 1997; Paulene et al. 2010; Hermens et al. 2013). New usage of virus removal filter where the target to be filtered is not proteins but rather viruses.

## Conclusions and outlook

Planova has been introduced into the industrial manufacturing processes in the last three decades. VF has become the de facto standard as a common unit operation as the most robust virus reduction technology for biological products, and Planova has made a significant contribution to ensure the viral safety. Based on the spinning technology of cuprammonium regenerated cellulose cultivated in Bemberg fiber and artificial kidney and basic research on phase separation, Planova is produced by the devisal of the original U-shaped spinning technique. Further, the development of the small virus-retentive VF (Planova20N) by deepening the phase separation technology and the innovative activities of the characterization of the VF have been attempted. Planova is made of highly hydrophilic cellulose hollow fiber and has a three-dimensional network structure consisting of interconnected void pores and capillary pores, designed to less breakage of the pore structure, which provides the advantage of low flux decay during filtration and high protein recovery while also providing high virus removability.

By strength in a deep understanding of technologies of phase separation, advanced control technology of membrane structure, and advanced characterization analysis of membranes using viruses, proteins and etc., we believe that VF can contribute to the future response to new needs for manufacturing biological products and the utilization of VF for new therapeutics such as gene therapy.

## References

[CR1] Adams B, Bak H, Tustian AD (2020). Moving from the bench towards a large scale, industrial platform process for adeno-associated viral vector purification. Biotechnol Bioeng.

[CR2] Adan-Kubo J, Tsujikawa M, Takahashi K, Hongo-Hirasaki T, Sakai K (2019). Microscopic visualization of virus removal by dedicated filters used in biopharmaceutical processing: impact of membrane structure and localization of captured virus particles. Biotechnol Prog.

[CR3] European Medicines Agency/CHMP/BWP P: *Guideline on virus safety evaluation of biotechnological investigation medicinal products EMEA/CHMP/BWP/398498/2005, 2008.7.24*

[CR4] European Medicines Agency/CHMP: *Guideline on plasma-derived medicinal products. CPMP/BWP/269/95 rev.4, 2009.2.19*

[CR5] Aranha H (2001). Viral clearance strategies for biopharmaceutical safety, part 1: general considerations. Biopharm.

[CR6] Aranha H (2001). Viral clearance strategies for biopharmaceutical safety, part 2: filtration for viral clearance. Biopharm.

[CR7] Ayano M, Sawamura Y, Hongo-Hirasaki T, Nishizaka T (2021). Direct visualization of virus removal process in hollow fiber membrane using an optical microscope. Sci Rep.

[CR8] Blümel J, Musso D, Teitz S, Miyabayashi T, Boller K, Schnierle BS, Baylis SA (2017) Inactivation and removal of Zika virus during manufacture of plasma-derived medicinal products. Transfusion 57(3pt2):790–796. 10.1111/trf.13873 10.1111/trf.13873 27731495

[CR9] Bogedain C, Maass G, Hörer M (1997) Filtration method for separation viruses. US 6479273B1

[CR10] Cao Y, Tan H (2006). Preparation and properties of microporous cellulose membranes from novel cellulose/aqueous sodium hydroxide solutions. J Appl Polym Sci.

[CR11] Cetlin D, Pallansch M, Fulton C, Vyas E, Shah A, Sohka T, Dhar A, Pallansch L, Strauss D (2018). Use of a noninfectious surrogate to predict minute virus of mice removal during nanofiltration. Biotechnol Prog.

[CR12] FDA/CBER: *Points to Consider in the Manufacture and Testing of Monoclonal Antibody products for Human Use 1997* 10.1097/00002371-199705000-00007 9181460

[CR13] Fisher AC, Kamga MH, Agarabi C, Brorson K, Lee SL, Yoon S (2019). The current scientific and regulatory landscape in advancing integrated continuous biopharmaceutical manufacturing. Trends Biotechnol.

[CR14] Furuya K, Murai K, Yokoyama T, Maeno H, Takeda Y, Murozuka T, Wakisaka A, Tanifuji M, Tomono T (2006). Implementation of a 20-nm pore-size filter in the plasma-derived Factor VIII manufacturing process. Vox Sang.

[CR15] Hamamoto R, Ito H, Hirohara M, Chang R, Hongo-Hirasaki T, Hayashi T (2018). Interactions between protein molecules and the virus removal membrane surface: effects of immunoglobulin G adsorption and conformational changes on filter performance. Biotechnol Prog.

[CR16] Hermens WTJMC, Smith JP March (2013) Removal of contaminating viruses from AAV preparations. World Intellectual Property Organization Patent 2013/036118A1

[CR17] Hongo-Hirasaki T, Yamaguchi K, Yanagida K, Okuyama K (2006). Removal of small viruses (parvovirus) from IgG solution by virus removal filter Planova 20N. J Membr Sci.

[CR18] Hongo-Hirasaki T, Komuro M, Ide S (2010). Effect of antibody solution conditions on filter performance for virus removal filter Planova TM 20N. Biotechnol Prog.

[CR19] Hongo-Hirasaki T, Yamaguchi K, Yanagida K, Hayashida H, Ide S (2011). Effects of varying virus-spiking conditions on a virus-removal filter Planova TM 20N in a virus validation study of antibody solutions. Biotechnol Prog.

[CR20] ICH.Q5A (R1): *Viral safety evaluation of biotechnology products derived from cell lines of human or animal origin 1997*

[CR21] Ide S, Tsurumi T, Nagashima H (1991) Filter membranes for physiologically active substances. Japanese Patent 3093821

[CR22] Ide S and Noda T (2000) Filter membranes for physiologically active substances. U.S. Patent 6797169

[CR23] Ide S, Ishizaki Y, Satoh S, Nakano H (2002) Effective Removal of Small Non-enveloped Viruses from Large Molecule. Biotherapeutic Products using New Virus Removal Filter, Planova 20N. CHI’s Blood Product Safety & TSE, February 4–7, 2002

[CR24] Iijima H, Iwata M, Inamoto M, Kamide K (1997). Phenomenological effects of solvent-casting conditions on pore characteristics of regenerated cellulose membranes. Polym J.

[CR25] Inamoto M, Miyamoto I, Hongo T, Iwata M, Okajima K (1996). Morphological formation of the regenerated cellulose membranes recovered from its cuprammonium solution using various coagulants. Polym J.

[CR26] Inouye M, Burnouf T (2020). The role of nanofiltration in the pathogen safety of biological products: an update. Curr Nanosci.

[CR27] Ittou M (2007) Bemberg business history. SEN’I GAKKAISHI 63(4):106–108. https://www.jstage.jst.go.jp/article/fiber/63/4/63_4_P_106

[CR28] Jorquera JI (2009). Flebogamma^®^ 5% DIF development: rationale for a new option in intravenous immunoglobulin therapy. Clin Exp Immunol.

[CR29] Junter GA, Lebrun L (2017). Cellulose-based virus-retentive filters: a review. Reviews Environ Sci Bio/technol.

[CR30] Kamide K, Iijima H, Matsuda S (1993). Thermodynamics of formation of porous polymeric membrane by phase separation I. Nucleation and growth of nuclei. Polym J.

[CR31] Kamide K and Manabe S (1985). Role of Microphase Separation Phenomena in the Formation of Porous Polymeric Membranes. “Material Science of Synthetic Membranes” ACS Symposium Series, No. 269, D. R. Lloyd, Ed., American Chemical Society, Washington, D. C., p197–228

[CR32] Kamide K, Iijima H, Shirataki H (1994). Thermodynamics of Formation of Porous Polymeric Membrane by Phase Separation Method II. Particle Simulation Approach by Monte Carlo Method and Experimental Observations for the Process of Growth of Primary Particles to Secondary Particles. Polym J 26(1):21–31. 10.1295/polymj.26.21

[CR33] Kamide K (1990) Thermodynamics of Polymer Solutions, Phase Equilibria and Critical Phenomena, Elsevier, Amsterdam

[CR34] Kapsch A, Farcet MR, Wieser A, Ahmad MQ, Miyabayashi T, Baylis SA, Blümel J, Kreil TR (2020). Antibody-enhanced hepatitis E virus nanofiltration during the manufacture of human immunoglobulin. Transfusion.

[CR35] Kesting RE (1990). The four tiers of structure in integrally skinned phase inversion membranes and their relevance to the various separation regimes. J Appl Polym Sci.

[CR36] Kesting RE (1985) Phase inversion membranes. ACS Symposium Series, No. 269, D. R. Lloyd, Ed., American Chemical Society, Washington, D. C., p131

[CR37] LaCasse D, Lute S, Fiadeiro M, Basha J, Stork M, Brorson K, Godavarti R, Gallo C (2016). Mechanistic failure mode investigation and resolution of parvovirus retentive filters. Biotechnol Prog.

[CR38] Liu S, Zeng J, Tao D, Zhang L (2010). Microfiltration performance of regenerated cellulose membrane prepared at low temperature for wastewater treatment. Cellulose.

[CR39] Lute S, Kozaili J, Johnson S, Kobayashi K, Strauss D (2020). Development of small-scale models to understand the impact of continuous downstream bioprocessing on integrated virus filtration. Biotechnol Prog.

[CR41] Manabe S, Kamata Y, Iijima H, Kamide K (1987). Some morphological characteristics of porous polymeric membranes prepared by “micro-phase separation method”. Polym J.

[CR42] Manabe S, Iwata M, Inoue M (1981) Japanese Patent 1556023; Japanese Patent 1556024; Japanese Patent 1625064; Japanese Patent 1434154; U.S. Patent 4581140

[CR43] Manabe S (1992) Virus removal membrane BMM (Planova, Mycocut). SEN’I GAKKAISHI 48(6):291–293. https://www.jstage.jst.go.jp/article/fiber1944/48/6/48_6_P291

[CR44] Manabe S (2003) Strategy against infections of virions and bovine spongiform encephalopathy (BSE) prion. J Mass Spectrom Soci Japan 51(1):146–152. https://www.jstage.jst.go.jp/article/massspec/51/1/51_1_146

[CR45] Mao Y, Zhou J, Cai J, Zhang L (2006). Effects of coagulants on porous structure of membrane prepared from cellulose in NaOH/urea aqueous solution. J Membr Sci.

[CR46] Ministry of Health and Welfare (2000): *Viral safety evaluation of biotechnology products derived from human or animal cell lines 2000.2.22*

[CR47] Parenteral Drug Association (PDA) (2005) Virus filtration Technical report No. 41. : PDA J. Pharm Sci. Technol. 59 (2 suppl TR41):8–42 15984110

[CR48] Paulene MQS, Gagnon P, Nichols G, Thorne BA (2010) Patent No. WO 2010148143

[CR49] Penaud-Budloo M, François A, Clément N, Ayuso E (2018). Pharmacology of recombinant adeno-associated virus production. Mol Therapy: Methods Clin Dev.

[CR50] Pollock J, Coffman J, Ho SV, Farid SS (2017). Integrated continuous production of recombinant therapeutic proteins. Biotechnol Prog.

[CR51] Roth NJ, Dichtelmüller HO, Fabbrizzi F, Flechsig E, Gröner A, Gustafson M, Jorquera JI, Kreil TR, Misztela D, Moretti E, Moscardini M, Poelsler G, More J, Roberts P, Wieser A, Gajardo R (2020). Nanofiltration as a robust method contributing to viral safety of plasma-derived therapeutics: 20 yearsʼ experience of the plasma protein manufacturers. Transfusion.

[CR52] Strauss D, Goldstein J, Hongo-Hirasaki T, Yokoyama Y, Hirotomi N, Miyabayashi T, Vavante D (2017). Characterizing the impact of pressure on virus filtration processes and establishing design spaces to ensure effective parvovirus removal. Biotechnol Prog.

[CR53] Tsurumi T, Osawa N, Hitaka H, Hirasaki T, Yamaguchi K, Manabe S, Yamashiki T (1990). Structure of cuprammonium regenerated cellulose hollow fiber (BMM hollow fiber) for virus removal. Polym J.

[CR54] Tsurumi T, Sato T, Osawa N, Hitaka H, Hirasaki T, Yamaguchi K, Hamamoto Y, Manabe S, Yamashiki T, Yamamoto N (1990). Structure and filtration performance of improved cuprammonium regenerated cellulose hollow fiber (improved BMM hollow fiber) for virus removal. Polym J.

[CR55] Tsurumi T (1991) The Latest Technology of Solution Spinning. SEN’I GAKKAISHI 47(10): 570–588. https://www.jstage.jst.go.jp/article/fiber1944/47/10/47_10_P570

[CR56] Wang S, Lu A, Zhang L (2016). Recent advances in regenerated cellulose materials. Prog Polym Sci.

[CR57] van de Witte P, Dijkstra PJ, van den Berg JWA, Feijen J (1996). Phase separation process in polymer solutions in relation to membrane formation. J Membrane Sci.

[CR58] Woods MA, Zydney AL (2014). Effects of a pressure release on virus retention with the Ultipor DV20 membrane. Biotechnol Bioeng.

[CR59] World Health Organization (2004). Guideline on viral inactivation and removal procedures intended to assure the viral safety of human blood plasma products. WHO Technical Report.

[CR60] Yamaguchi K, Miyagawa E, Takahashi H, Miyazaki T, Ikeda H (2007). Electron microscopic estimation of removal of parvovirus B19 (HPVB19) by nanofiltration with a novel filter membrane. J Membr Sci.

[CR61] Yamamoto A, Hongo-Hirasaki T, Uchi U, Hayashida H, Nagoya F (2014). Effect of hydrodynamic forces on virus removal capability of Planova filter. Aich Journal.

[CR62] Yokogi M, Satoh S (1998). Development and application of virus removal filter ‘PLANOVA’. Pharm Tech Japan.

[CR63] Yue C, Teitz S, Miyabayashi T, Boller K, Lewis-Ximenez LL, Baylis SA, Blümel J (2019). Inactivation and removal of chikungunya virus and mayaro virus from plasma-derived medicinal products. Viruses.

[CR64] Zhang J, Shao H, Wu C, Hu X (2001). Formation and characterization of cellulose membranes from *n*-methylmorpholine-*N*-oxide solution. Macromol Biosci.

[CR65] Zhang J, Conley L, Pieracci J, Ghose S (2017). Pool-less processing to streamline downstream purification of monoclonal antibodies. Eng Life Sci.

